# The data‐index: An author‐level metric that values impactful data and incentivizes data sharing

**DOI:** 10.1002/ece3.8126

**Published:** 2021-10-13

**Authors:** Amelia S. C. Hood, William J. Sutherland

**Affiliations:** ^1^ Conservation Science Group, Department of Zoology University of Cambridge Cambridge UK; ^2^ Biosecurity Research Initiative at St Catharine's (BioRISC), St Catharine's College University of Cambridge Cambridge UK

**Keywords:** author‐level metrics, bibliometrics, data citation, data metrics, data sharing, dataset repositories, FAIR research data, h‐index, open science

## Abstract

Author‐level metrics are a widely used measure of scientific success. The h‐index and its variants measure publication output (number of publications) and research impact (number of citations). They are often used to influence decisions, such as allocating funding or jobs. Here, we argue that the emphasis on publication output and impact hinders scientific progress in the fields of ecology and evolution because it disincentivizes two fundamental practices: generating impactful (and therefore often long‐term) datasets and sharing data. We describe a new author‐level metric, the data‐index, which values both dataset output (number of datasets) and impact (number of data‐index citations), so promotes generating and sharing data as a result. We discuss how it could be implemented and provide user guidelines. The data‐index is designed to complement other metrics of scientific success, as scientific contributions are diverse and our value system should reflect that both for the benefit of scientific progress and to create a value system that is more equitable, diverse, and inclusive. Future work should focus on promoting other scientific contributions, such as communicating science, informing policy, mentoring other scientists, and providing open‐access code and tools.

## Graphical abstract

Our current value system in academia centers around publications. This hinders scientific progress as it disincentivizes fundamental practices, such as generating and sharing data. Here, we describe a new author‐level metric (the data‐index) which values these practices and promotes them as a result. The data‐index is designed to complement other indices of scientific success, and create a broader perspective of scientific impact.

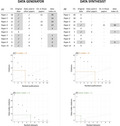

## 1 INTRODUCTION

Despite many concerns, measuring scientific success with author‐level metrics has become widely used, including in funding and job allocations (Hicks & Wouters, 2015; Mingers & Leydesdorff, 2015). The h‐index is the most common means of comparison; it combines the publication output (number of publications) and research impact (as number of citations) of authors (Hirsch, 2005). Despite its many flaws, the h‐index has flourished because it is a simple and easily calculable measure of scientific impact. Scientists have designed similar metrics that address some of its flaws (Gasparyan et al., 2018; Mingers & Leydesdorff, 2015), such as encouraging unwarranted self‐citations (Bartneck & Kokkelmans, 2011; Senanayake et al., 2015), but these metrics are not yet as widely adopted as the h‐index. Such flaws are not the focus of this paper. Here, we argue that any value system that predominantly focuses on publications will hinder scientific progress.

We need a value system that captures our wider contributions to science, both for the benefit of scientific progress and to create a value system that is more equitable, diverse, and inclusive (Davies et al., 2021). These contributions include communicating science, informing policy, mentoring other scientists, getting sponsorship, facilitating collaborations, providing open‐access code and tools, and generating useful datasets with long‐term value. Some author‐level metrics have been designed with this aim; Altmetrics quantifies the online impact of publications (Sud & Thelwall, 2014), and Barres (2013) suggested a metric for mentoring quality. However, many other important contributions are not yet quantified.

Here, we present a new author‐level metric, the data‐index, designed to value dataset output (number of datasets) and research impact (number of data‐index citations) and complement other metrics of scientific success. Ignoring this dimension of scientific success results in researchers being incentivized to produce impactful publications rather than impactful datasets with long‐term value. This is particularly detrimental in ecology and evolutionary biology, where datasets produced in long‐term studies contribute disproportionately both scientific understanding and to policy (Hughes et al., 2017; Mills et al., 2015). Incentivizing long‐term studies is especially important because they are expensive and arduous, and pressure to publish frequently is one of the key systemic barriers that hinders efforts to conduct them (Kuebbing et al., 2018). Therefore, a value system that better rewards generating impactful datasets, such as through long‐term experiments, would benefit research in ecology and evolution.

Undervaluing dataset output and impact also disincentivizes data sharing. Researchers that share their data are frequently not listed as coauthors on the resulting publications. This means that the researcher's h‐index is largely unaffected; if they are likely to lack what they see as sufficient credit for their contribution, they may be reluctant to share data (Ewers et al., 2019). This issue reduces opportunities for reanalysis. It is particularly prevalent in evidence synthesis, where data generators may have additional concerns that their publication will be overlooked if a synthesis paper becomes available (Patsopoulos et al., 2005; Poisot et al., 2019), and indeed, papers cited by formal review articles can experience a dramatic loss in future citations (McMahan & McFarland, 2021). In fact, synthesists have limited success in retrieving data when requested, with an estimated success rate of less than 50% if the researcher is not known personally (Côté et al., 2013; Vines et al., 2014). Synthesists contend that the citation that they give the generator's paper is sufficient credit, that generating the data does not meet authorship requirements, or that listing all generators as coauthors would be infeasible (Ewers et al., 2019). According to the Vancouver recommendations for authorship, collecting data alone does not justify authorship (ICMJE, 2019). Synthesis papers provide robust evidence for scientific theories and should be cited if relevant, and citing all of the papers within them is usually infeasible. Therefore, we clearly need a new approach. Better incentives for sharing data would also increase scientific reproducibility and save costs by avoiding the unnecessary duplication of results (Grainger et al., 2020; Piwowar et al., 2011; Wilkinson et al., 2016). The importance of data sharing is now widely recognized, and journals are increasingly mandating public data archiving (Mislan et al., 2016). However, the majority of authors in ecology and evolution (estimated 64% by Roche et al. (2015)) archive their data in a way that prevents reuse. This demonstrates the need for an improved value system that better rewards sharing data in a useful manner.

Our call to better reward authors for producing and sharing impactful data echoes the calls of others before us, including those that developed the FAIR (Findable, Accessible, Interoperable, Reusable) Guiding Principles for data management (Ewers et al., 2019; Konkiel, 2020; Wilkinson et al., 2016). In the last decade, huge efforts have been made to increase the recognition of datasets, including the development of dataset repositories, the largest of which are DataCite and Thomson Reuters Data Citation Index (Arend et al., 2020; Cousijn et al., 2019; Konkiel, 2020; Pavlech, 2016). These repositories, respectively, store 21.8 and 10.3 million datasets as first‐class research outputs (“Data Citation Index,” 2021, “DataCite,” 2021). Datasets are given PIDs (i.e. persistent identifiers such as DOIs), citations of these datasets are tracked, and guidelines for citing data have been developed (Cousijn et al., 2018, 2019). Citations are well‐suited to showcase research impact as they are the most widely understood indicator for data, and these repositories have greatly increased the recognition of datasets (Konkiel, 2020). However, researchers are yet to be meaningfully recognized for their contributions; two recent workshops with 70 and 32 stakeholders recommended prioritizing developing metrics as a way to give credit to researchers for generating and sharing data (Federer, 2020; Pierce et al., 2019). Here, we present an author‐level metric based on dataset characteristics with the aim of promoting data sharing and increasing the recognition of authors that produce impactful data. We discuss how it could be implemented by building on the existing frameworks of dataset repositories and provide user guidelines.

## 2 THE DATA‐INDEX

**FIGURE 1:**
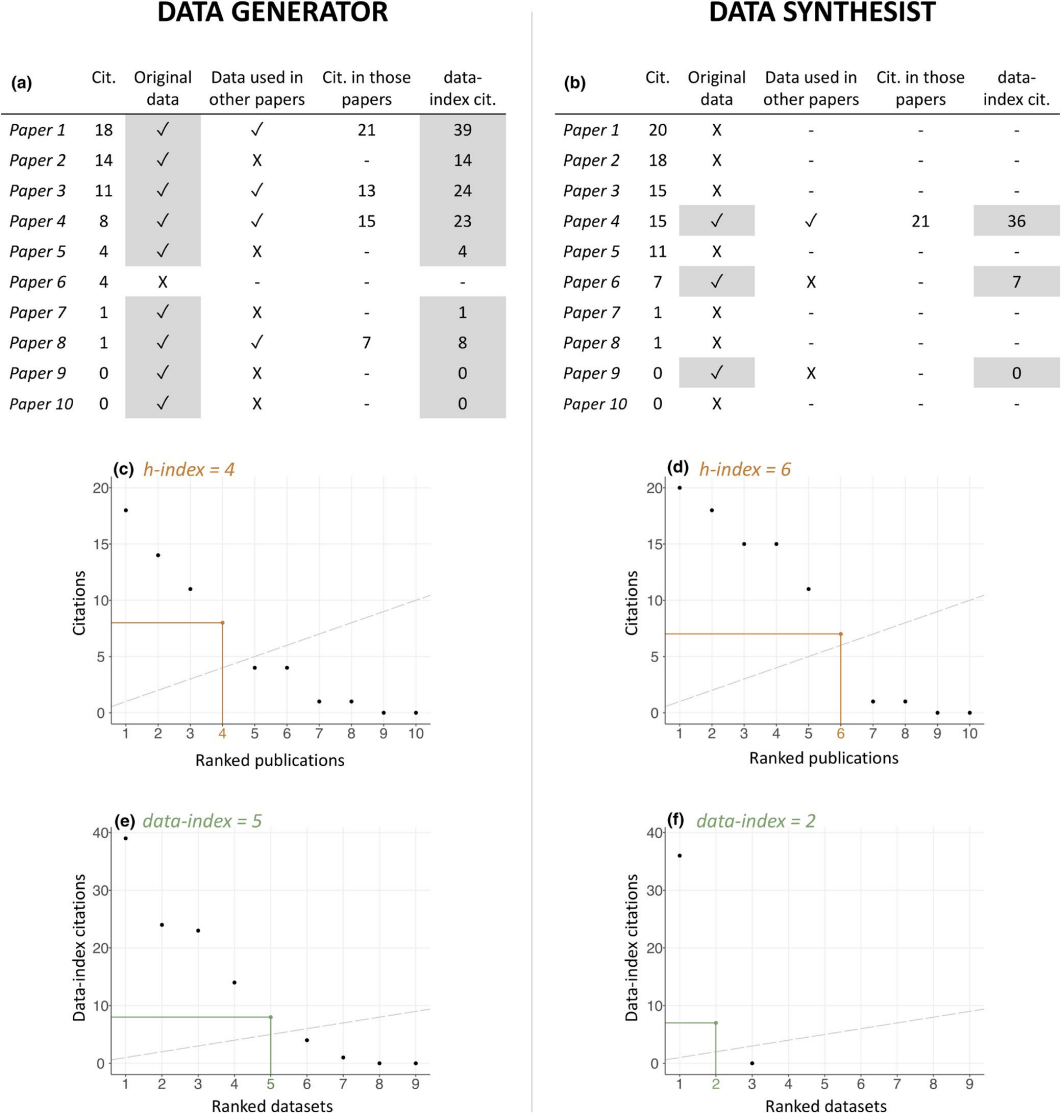
A composite figure with a hypothetical example that shows how the h‐index and data‐index for a data generator (i.e. someone who generates data, e.g. by conducting experiments) and data synthesist (i.e. someone who synthesizes research, e.g. through systematic reviews) at a similar career stage might differ. The h‐index is equal to the number of publications (*n_p_*) that have *n*_*p*_ or more citations, whereas the data‐index is equal to the number of datasets (*n_d_*) that have *n_d_* or more data‐index citations. For both indices, publications or datasets are considered the same whether the author was primary author or coauthor. (a, b) Tables showing example data used to calculate the h‐index and data‐index shown in plots (c–f). (a, b) Papers with original data (highlighted in gray) are the only ones included in the calculation of the data‐index. Scatterplots with (c, d) publications ranked by citations to calculate the h‐index and (e, f) datasets ranked by data‐index citations to calculate the data‐index. Dashed lines show identity lines, and colored lines show the final publication/dataset used to calculate the index value, which is also colored. In this hypothetical example, the data generator has a lower h‐index (4) than the data synthesist (6), but a higher data‐index (6 vs. 2). *Cit*. is an abbreviation for citation

**FIGURE 2:**
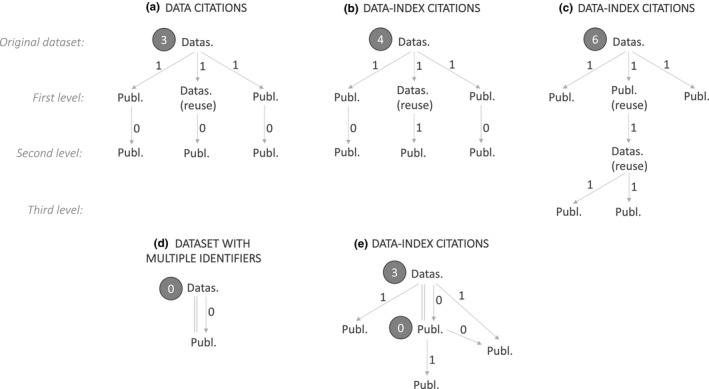
Diagrams showing that (a) data citations are calculated by summing the first‐level citations of a dataset, whereas (b, c) data‐index citations are calculated by summing the first‐level citations of a dataset or publication that contains an original dataset and any higher‐level citations of datasets or publications that have reused data from the original dataset or publication. (d, e) In cases where the same dataset has multiple identifiers (e.g. if the dataset has a unique identifier and a publication describing it has a different unique identifier), existing citation mapping software can be used to automatically group them and therefore avoid the same dataset being double‐counted; parallel lines show datasets and publications that are grouped. Abbreviations are as follows: *Datas*. = dataset, *Publ*. = publication, *Datas*.*/Publ. (reuse)* = dataset or publication that has reused data from the original dataset. Arrows show the direction of citation, and numbers in black show the value this citation gives to calculating the citation score of the original dataset. White numbers in gray circles show the (a) data citation and (b–e) data‐index citation scores of the datasets beside them. Citation levels for (a–c) are shown on the left

The data‐index is calculated the same way as the h‐index, but original datasets are ranked in order of their data‐index citations rather than publications being ranked in order of publication citations (Figure 1). An author's data‐index is equal to the number of datasets (*n*) that they have published (as primary or coauthor) that have *n* or more *data‐index citations*. Data‐index citations differ from *data citations*, which are calculated by summing the first‐level citations of a dataset (Figure 2(a)) (Cousijn et al., 2018). Data‐index citations are calculate by summing first‐ and higher‐level citations (i.e. citations of datasets or publications that have cited the original dataset), but higher‐level citations are only counted from datasets or publications that have reanalyzed the original dataset (Figure 2(b,c)). This means that the generators of the original dataset gain data‐index citations anytime their data are cited, regardless of whether they have authored the datasets or publications that have reused their data. Though it is possible that data‐index citations from the second level or higher are for datasets that are not reusing data from the original dataset (Figure 2(c)) (for example, if a subset of data that did not include the original dataset were selected from the first‐level dataset), we do not think this will be a common issue as data sharing rates are currently low. This may need to be revised if data sharing practices change. Figure 1 shows a hypothetical example of how a data generator and synthesist at a similar career stage might differ. The generator has a lower h‐index than the synthesist because their publications have fewer citations, but their data‐index is higher because they have more original datasets, many of which have been reused.

The data‐index measures research impact (citations) rather than reuse (the number of times a dataset has been reused). For example, a dataset that is used to inform an important theory in a single highly cited paper will be considered impactful (as there are many first‐level citations) even if it is not reused. However, dataset reuse (and therefore data sharing) is promoted by the data‐index as the data‐index citations of a dataset will increase through higher‐level citations anytime the dataset is reused and cited (Figure 2). Though the impact (citations) of a paper could be unrelated to the data within it, we think that this will be in a minority of cases. Therefore, the data‐index approximates how important a dataset is in informing concepts that are important within the scientific community, rather than how reusable the dataset is, but it does promote data reuse and therefore sharing.

## 3 IMPLEMENTING THE DATA‐INDEX

Calculating the data‐index is not yet automated. Dataset repositories are developing the ability to automate calculating data‐citations of the datasets in their repositories (Cousijn et al., 2019; Pavlech, 2016), and many of these datasets can be automatically linked to authors in Web of Science (Mongeon et al., 2017). We would need to build on this existing framework to include higher‐level citations to automate calculating data‐index citations. Another technical consideration is cases where the same original dataset is duplicated, for example, if there is a dataset with a PID and a data descriptor paper with a separate PID. Objects with different PIDs can be grouped and linked (e.g. via citation mapping software (“Connected Papers,” 2021), and citation repositories are adept at doing this (Cousijn et al., 2021; Groth et al., 2020). This functionality could be used when calculating data‐index citations so that the same dataset is not counted multiple times (Figure 2(d,e)).

A further consideration is datasets that do not have their own PIDs as they are only presented as part of publications. We recommend that these publications (*data studies*) are considered as original datasets because publishing datasets as first‐order scientific objects is a recent practice (Robinson‐García et al., 2016). Furthermore, directly citating datasets are not widely practiced (Federer, 2020) (e.g. 88% of Data Citation Index datasets have no citations (Robinson‐García et al., 2016)) and even well‐intentioned authors that want to cite datasets and publications in their papers, as recommended by the Joint Declaration of Data Citation Principles (Cousijn et al., 2018), may be limited by journal restrictions on reference list length. We echo the recommendation that datasets should be published as independent scientific objects to make research FAIR (Wilkinson et al., 2016), but author‐level data metrics would be inaccurate if data studies were excluded because of current and historic data‐publishing practices. Dataset repositories already include some data studies (e.g. there are 1.3 million data studies on the Data Citation Index (“Data Citation Index,” 2021)), but wider coverage is needed to calculate complete author‐level data metrics.

A final consideration is what the unit of a dataset is, and defining this is not as straightforward as defining a publication (Konkiel, 2020). It raises questions such as.

- Is there a minimum or maximum size for a dataset?
- Are two figures one dataset or two datasets?
- When does an updated dataset become a new dataset?
- Does combining datasets make a new dataset?

Answering these questions is beyond the scope of this paper as they should be addressed by groups of stakeholders, including: publishers, funders, librarians, repository administrators, open science organizations and researchers from across the disciplines. Stakeholders and data‐citation experts have started this process, and developed guidelines and technical solutions to several of these issues (Cousijn et al., 2018; Federer, 2020). For example, data outputs can be grouped into single units, and the origin of datasets can be tracked in data repositories (e.g. updated datasets are labeled “isNewVersionOf”) (Groth et al., 2020). We recommend a baseline of one dataset per publication, which users could deviate from according to data‐citation experts' advice as it develops.

## 4 GUIDELINES FOR USE

As with all author‐level metrics, the data‐index should be used with caution. Scientific value cannot be accurately or fairly quantified by multiple indices, but multiple indices create a more balanced view than single ones (Hicks & Wouters, 2015). We refer the reader to the Leiden Manifesto, which gives ten principles to guide research evaluation (Hicks & Wouters, 2015). We highlight four specifically: Indicators should not substitute expert assessment; research performance should be measured against the aims of the institution or researcher; variation by field in publication and citation practices should be accounted for (Kokko & Sutherland, 1999); and false precision should be avoided.

The similarity of the h‐index and the data‐index has advantages and disadvantages. The data‐index shares many of the limitations of the h‐index (Gasparyan et al., 2018; Mingers & Leydesdorff, 2015), including using citation counts, which are biased (Davies et al., 2021). Both are liable to gaming by increasing unwarranted self‐citations, and the h‐index variants that correct for this could be adapted to the data‐index (Bartneck & Kokkelmans, 2011; Senanayake et al., 2015). Using corrected and uncorrected metrics would give a more comprehensive view, as high rates of self‐citation can be legitimate if researchers are pioneers in their field. A further concern is that both indices consider the contributions of all authors as equal, which can result in authors in successful groups having higher index scores than their counterparts in less successful groups, simply because they have coauthored many papers/datasets despite giving little input. Evaluators should consider that the data‐index reinforces this bias. On balance, we think that the similarity between the h‐index and data‐index is beneficial, however, because its familiarity makes the limitations transparent. Citation is a concept that is well understood and valued by many in academia, and most researchers are aware of the benefits and limitations of using it for evaluations (Konkiel, 2020). Evaluators must exercise caution when using author‐level metrics, which should be revised and updated as necessary (Hicks & Wouters, 2015).

## 5 METRICS FOR OTHER CHARACTERISTICS OF DATASETS

The data‐index calculates dataset output and research impact rather than the other characteristics of datasets, such as data usage (e.g. downloads), data reuse (e.g. times a dataset it reused), data quality (e.g. accuracy and completeness), social impact (e.g. use in informing policy), and whether the data are open access or not (Konkiel, 2020). Indicators for these characteristics are limited, and author‐level metrics have not yet been developed (Konkiel, 2020). We have focussed on dataset output and impact as these characteristics reflect our current value system (publication output and impact via the h‐index), and they encompass several of the most important features of data production. Some of the other characteristics will probably also be reflected in the data‐index. For example, greater data usage and reuse would likely correlate with greater data impact, but data quality is not likely to be reflected in the data‐index; publications with higher quality data—in terms of sample size, variance (Barto & Rillig, 2012) and replicability (Yang et al., 2020)—are not cited more than lower quality studies. Publications with open‐access data are more highly cited than those without, but the mechanism for this is not yet known (Colavizza et al., 2020). Metrics that measure these characteristics directly could be quantified in other complementary indices. For example, The Google Scholar Public Access Index (Van Noorden, 2021), which measures the proportion of an author's papers that are required by their funders to be open access are actually freely available online, could be adapted to datasets to measure data openness.

## 6 DISCUSSION

The data‐index addresses two major issues in the fields of ecology and evolution: that generating impactful datasets, such as long‐term datasets, is undervalued, and that sharing data is disincentivized (Federer, 2020; Mills et al., 2015). Others have suggested changing authorship categories (Ewers et al., 2019) or using a standardized way to allocate coauthor contributions (e.g. via the CRediT system (Ding et al., 2021)) to better value data generators and address these issues. These approaches can be used in addition to the data‐index, but they are fundamentally different as the data‐index does not discriminate between the roles of coauthors in terms of their contribution to generating useful datasets. It does not specifically target data collectors, but all authors that contribute to a dataset, whether by collecting or curating the data. The data‐index can be implemented retrospectively, where the other methods cannot, and it complements our existing system of using author‐level metrics. The data‐index answers calls from across the scientific and publishing communities to develop metrics that give credit to researchers for generating and sharing data (Federer, 2020; Pierce et al., 2019).

Our current value system (the h‐index) is an oversimplification that hinders scientific progress and impact. Scientific contribution is diverse, and we need metrics that better value this diversity, both for the benefit of scientific progress and to create a value system that is more equitable, diverse, and inclusive (Davies et al., 2021). The data‐index should be used to complement other author‐level metrics and create a broader perspective of scientific impact. More work should be done to promote other aspects of scientific success, such as science communication (Sud & Thelwall, 2014), informing policy, mentoring scientists (Barres, 2013), getting sponsorship, facilitating collaborations, or providing open‐access materials. Like many ecological and evolutionary processes, scientific success is multidimensional, and we must create a system that better values that.

## CONFLICT OF INTEREST

None declared.

## AUTHOR CONTRIBUTIONS

**Amelia S. C. Hood:** Conceptualization (lead); methodology (lead); visualization (lead); writing–original draft (lead); writing–review and editing (equal). **William J. Sutherland:** Conceptualization (supporting); methodology (supporting); writing–review and editing (equal).

## Data Availability

The authors confirm that all of the data are available in the article.
